# Maternal Vitamin D Level Is Associated with Viral Toll-Like Receptor Triggered IL-10 Response but Not the Risk of Infectious Diseases in Infancy

**DOI:** 10.1155/2016/8175898

**Published:** 2016-05-19

**Authors:** Sui-Ling Liao, Shen-Hao Lai, Ming-Han Tsai, Man-Chin Hua, Kuo-Wei Yeh, Kuan-Wen Su, Chi-Hsin Chiang, Shih-Yin Huang, Chuan-Chi Kao, Tsung-Chieh Yao, Jing-Long Huang

**Affiliations:** ^1^Community Medicine Research Center, Chang Gung Memorial Hospital at Keelung, Keelung 204, Taiwan; ^2^Department of Pediatrics, Chang Gung Memorial Hospital at Keelung, Keelung 204, Taiwan; ^3^Division of Pulmonology, Department of Pediatric, Chang Gung Memorial Hospital and Chang Gung University, College of Medicine, Taoyuan 333, Taiwan; ^4^Division of Allergy, Asthma, and Rheumatology, Department of Pediatrics, Chang Gung Memorial Hospital and Chang Gung University, College of Medicine, Taoyuan 204, Taiwan; ^5^Department of Obstetrics and Gynecology, Chang Gung Memorial Hospital at Keelung, Keelung 204, Taiwan

## Abstract

Reports on the effect of prenatal vitamin D status on fetal immune development and infectious diseases in childhood are limited. The aim of this study was to investigate the role of maternal and cord blood vitamin D level in TLR-related innate immunity and its effect on infectious outcome. Maternal and cord blood 25 (OH)D level were examined from 372 maternal-neonatal pairs and their correlation with TLR-triggered TNF-*α*, IL-6, and IL-10 response at birth was assessed. Clinical outcomes related to infection at 12 months of age were also evaluated. The result showed that 75% of the pregnant mothers and 75.8% of the neonates were vitamin deficient. There was a high correlation between maternal and cord 25(OH)D levels (*r* = 0.67, *p* < 0.001). Maternal vitamin D level was inversely correlated with IL-10 response to TLR3 (*p* = 0.004) and TLR7-8 stimulation (*p* = 0.006). However, none of the TLR-triggered cytokine productions were associated with cord 25(OH)D concentration. There was no relationship between maternal and cord blood vitamin D status with infectious diseases during infancy. In conclusion, our study had shown that maternal vitamin D, but not cord vitamin D level, was associated with viral TLR-triggered IL-10 response.

## 1. Introduction

Vitamin D has been shown to play an important role in both the innate and adaptive immune system. In vitro studies have demonstrated vitamin D to correlate with alterations of several cytokines such as IL-4, IL-5, IL-6, IL-10, IL-13, and interferon *γ* [1–3]. Hence, as a potent immune modulator, vitamin D was shown to be associated with childhood asthma and allergic diseases [4–6]. In addition to allergic diseases, vitamin D also partakes a potential role in airway inflammation, thus, strongly linked with acute infectious illness such as upper or lower respiratory tract infections, sepsis, and hospitalization [7–10]. Given the important immune modulatory role of vitamin D, the link between Toll-like receptors- (TLRs-) mediated innate immunity and vitamin D deserves in-depth investigation. Studies have shown vitamin D to downregulate TLR expression in order to dampen immune cytokine response in multiple basic science models [11–13]. In contrast, some reports have shown treatment with vitamin D to result in increased TLR activation. In a cohort study of 225 infants, higher 25(OH)D_3_ level at 6 months was associated with greater cytokine responses to TLR ligands [14–16]. Thus, despite a wide variety of studies that acknowledged the immunomodulatory role of vitamin D, the results have been conflicting. Furthermore, very few studies address the impact of prenatal vitamin D status on TLR-related innate immune response in neonatal infants. Because of the apparent importance of vitamin D in immune development, we aimed to investigate the effect of maternal and/or cord vitamin D level on TLR-triggered cytokine response in neonates at time of birth and disease outcome in early childhood. We focused attention on neonatal innate immunity since early life events appear to have a critical influence on the ultimate pattern of immune maturation. In addition, we sought to investigate whether maternal blood and cord blood vitamin D correspond in their association with TLR-related innate immunity.

## 2. Methods

### 2.1. Study Population

Data for this analysis came from an ongoing prospective birth cohort study called the PATCH (The Prediction of Allergy in Taiwanese Children). The Chang Gung Ethics Committee approved the study, and informed consent was obtained from the parents/legal guardians of the neonates. Pregnant women undergoing routine prenatal exam were approached randomly by a study nurse and invited to join our research program. All mothers and their offspring were enrolled upon agreement, but those born under the gestational age of 37 weeks, had major congenital anomaly, or were suspicious of congenital infections were subsequently excluded from this analysis. The result from this study comprised the first 372 eligible mother-neonatal pairs. A baseline questionnaire survey was conducted at birth to obtain parental information such as demographic characteristics, medical and obstetric history, and smoking exposure history. Standardized questionnaires on atopic heredity, environmental factors, infection, and allergic diseases were answered at 2, 4, 6, and 12 months and every year thereafter. Infants were defined as ever having lower respiratory tract infection (bronchiolitis, pneumonia, and/or croup) if there was a diagnosis from a health care professional, and the infant either had been hospitalized or received medical treatment. Other infections such as infectious enteritis and urinary tract infection were also obtained from medical records with physicians' diagnosis. By the time of analysis, 321 children included in this study were at least 1 year of age and had adequate follow-up data.

#### 2.1.1. Sample Collection, Cell Culture, and TLR Ligands Stimulation

The details of our experimental procedures have been published previously [17]. Briefly, maternal blood was obtained during third gestation and umbilical cord blood collected at the time of delivery. Mononuclear cells were isolated and stimulated with TLR ligands. These included synthetic bacterial lipoprotein (PAM3csk4) that is recognized by TLR1-2; a synthetic analog of double stranded RNA for TLR3; ultrapure LPS for TLR4; and R848, which is activated via the TLR7/TLR8 signaling pathway. As a positive control, cells were treated with the NF-*κ*B activator phytohemagglutinin (Murex Pharmaceuticals) at 4 *μ*g/mL in R10-FBS. To determine TLR responses, 3 × 10^5^ PBMCs in 100 *μ*L R10-FBS were added to each of the media or ligands (in duplicate), containing wells and incubated at 37°C for 20 h with 5% CO_2_. All assay preparations were performed using sterile technique in a laminar flow hood. The concentrations of the ligands used for this experiment are as follows: 10 *μ*g/mL of PAM3csk4, 10 *μ*g/mL of poly(I:C) directly administered, 20 ng/mL of LPS, and 10 *μ*g/mL of R848 (InvivoGen, San Diego, CA).

#### 2.1.2. Measurement of Cytokines

TNF-*α*, IL-10, and IL-6 levels in culture supernatants were determined by enzyme-linked immunosorbent assays according to the manufacturer's instructions (ELISA; R&D systems, MN). The detection limits were 15.6 pg/mL for TNF-*α*, 3.12 pg/mL for IL-6, and 7.8 pg/mL for IL-10.

#### 2.1.3. Serum 25(OH)D Measurement

Serum samples obtained from the pregnant mother and cord blood were stored frozen in aliquotsat −80°C until analysis. Serum 25(OH)D levels were measured by Elecsys Vitamin D total assay (Roche Diagnostics, Mannheim, Germany). This method is a new automated electrochemiluminescence-based assay that measures both the 25(OH)D_2_ and 25(OH)D_3_ as total 25(OH)D level. Results from this assay have shown close agreement to other well-established methods such as liquid-chromatography tandem mass spectrometry (LC-MS/MS) [18].

### 2.2. Statistical Methods

Spearman's rank correlation test was performed to analyze the correlation between maternal and cord blood 25(OH)D level. Since the concentrations of 25(OH)D and cytokines were not normally distributed, values were logarithmically transformed as continuous variables in the statistical models. Regression analysis was used to determine the relation between maternal/cord blood 25(OH)D concentration and TLR-induced cytokine response as continuous variables. Association between serum vitamin D level and binary outcomes (bronchiolitis, pneumonia, croup, infectious enteritis, and urinary tract infection) was analyzed by using logistic regression. Models were adjusted for gestational age, gender, birth body weight, mode of delivery, maternal allergy, and season of birth. All statistical analysis was carried out using IBM SPSS Statistics Version 20 (Armonk, NY).

## 3. Result

### 3.1. Subject and Demographic Data

**Figure 1:**
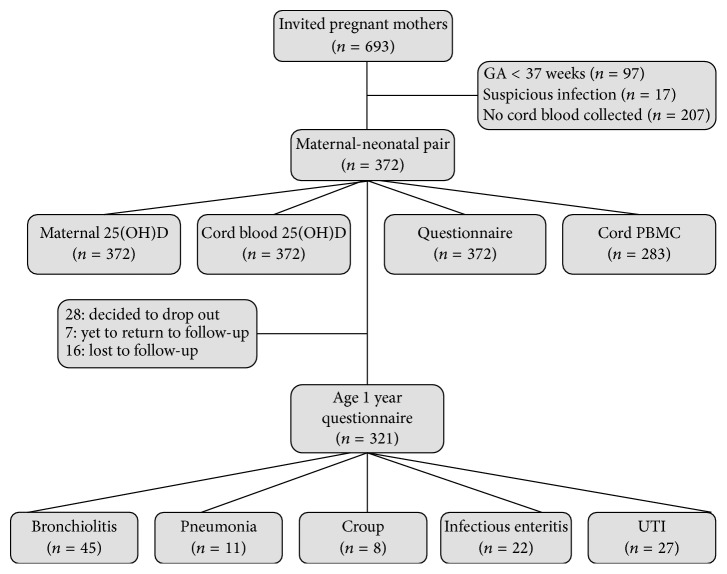
Flowchart of the analyzed maternal-neonatal pairs: demonstrating the number of study participants after consideration of available maternal and cord 25(OH)D measurements, cell culture data, and questionnaire information.

Characteristics of the mother-neonatal pairs are summarized in Table 1. Mean maternal age was 29.4 years. The incidence of maternal allergy was 34.8%, compatible with that of the general population. All neonates included in this analysis were above gestational age of 37 weeks with adequate birth body weight. Slightly more babies were delivered during the season of spring (28,8%). Of the original 372 maternal-neonatal pairs, fifty-one participants either were lost to follow-up, refused to further participate, or had yet to return. By the age of 12 months, 321 infants with complete questionnaires and medical records were available for analysis. Detailed number of participants and test samples are listed in Figure 1.

### 3.2. Maternal and Cord Vitamin D Levels

**Figure 2:**
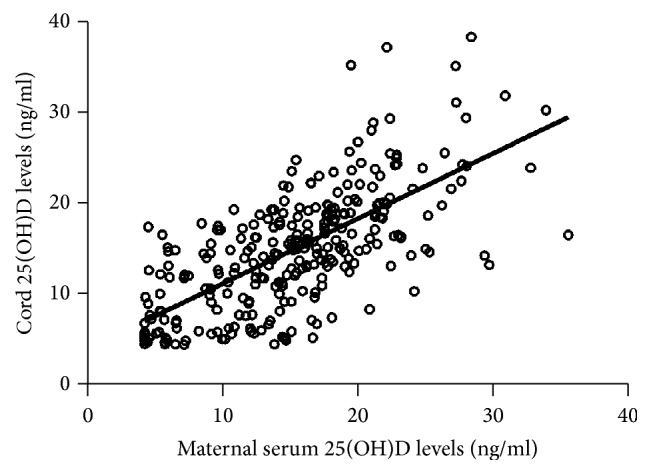
Correlation between maternal and cord blood 25(OH)D levels (ng/mL).

The median maternal blood 25(OH)D concentration was 15.18 ng/mL (interquartile range (IQ): 10.85–19.10 ng/mL), and the median cord blood 25(OH)D was 14.80 ng/mL (IQ: 10.02−18.86 ng/mL). Of the 372 maternal participants, 280 (75%) had 25(OH)D levels less than 20 ng/mL (considered deficient), and 71 (19%) had levels between 20 and 30 ng/mL (considered insufficient). An even higher percentage of vitamin D deficiency was found in the neonatal cord blood, with 279 (75.8%) having levels less than 20 ng/mL, and 55 (15%) between 20 and 30 ng/mL. There was a high correlation between maternal and cord 25(OH)D levels (*r* = 0.67, *p* < 0.001; Figure 2).

### 3.3. Association of Maternal and Cord Vitamin D Level with TLR-Stimulated Cytokine Response

Because the distribution of most cytokine levels and vitamin D concentrations was highly skewed (data not shown), we used natural log-transformed 25(OH)D and cytokine levels for correlation analysis. The result showed significant inverse correlation between maternal 25(OH)D level and IL-10 response to TLR3 and TLR7-8 stimulation (*p* = 0.007 and *p* = 0.008, resp.) in cord blood mononuclear cells. The result still remained significant after adjusting for potential confounding factors (*p* = 0.004 for TLR3 and *p* = 0.006 for TLR7-8) (Table 2(a)). However, neonatal cord 25(OH)D concentration was not associated with any of the TLR-triggered cytokine productions (Table 2(b)). Preliminary analysis was also performed on maternal innate immune function; the result showed no correlation between maternal vitamin D status and cytokine response to TLR ligands in maternal mononuclear cells (Supplement 1) (see Supplementary Material available online at http://dx.doi.org/10.1155/2016/8175898).

### 3.4. Vitamin D Level and Clinical Outcome

By the age of one year, 321 infants had their medical records reviewed and completed the questionnaires administered at 6 and 12 months of age. Analysis was made to investigate whether maternal or cord blood vitamin D status was associated with lower respiratory tract infection (bronchiolitis, pneumonia, and croup), infectious enteritis, and urinary tract infection at 1 year of age. The results, summarized in Table 3, showed no significant association between maternal vitamin D status and the incidence of infection during the first year of life. Cord blood vitamin D level was also not correlated with any of the infectious disorders by 12 months of age.

## 4. Discussion 

The result from our study showed that maternal, but not cord blood vitamin D level, was associated with TLR-3 and TLR7-8 triggered IL-10 response. To our knowledge, this is the first cohort study to simultaneously assess the effect of both maternal and cord blood vitamin D status on TLR-related immune response and various infectious diseases during infancy. Despite extensive investigations on vitamin D, its role in neonatal immune development and health outcome remains inconsistent. The reasons for various conflicting results might be due to differences in the study designs, age of the study population, definition of clinical outcomes, and also disparities in the assessment of vitamin D concentration (use of maternal blood or cord blood). Our result suggested that the latter might be an important issue to consider, because despite a strong correlation between maternal and cord vitamin D level, our study had observed a distinction between maternal and cord vitamin D status in their association with TLR-triggered cytokine response. Thus, when addressing the impact of prenatal vitamin D status on various outcomes, it might be important to consider that vitamin D levels in the pregnant mothers and cord blood might not always correspond uniformly. Similar observations were noted in few other studies that concurrently assessed maternal and cord vitamin D concentrations. In the study of Weisse et al., although both maternal and cord blood vitamin D levels were associated with clinical food allergy, only maternal 25(OH)D_3_ was associated with an increase in allergen sensitization [19]. Another recent publication had demonstrated that although low levels of vitamin D in the cord blood were associated with higher airway resistance in childhood, maternal vitamin D level was not related to the children's airway resistance [20].

The relationship between vitamin D and IL-10 has been established considerably. Our result had observed a negative correlation between maternal vitamin D concentration and TLR-induced IL-10 response. In contrast to our study, Vijayendra Chary et al. had observed lower cord blood IL-10 level in vitamin D deficient or insufficient subjects. In vitro studies have also shown direct vitamin D supplementation in culture human cells to upregulate IL-10 secretion [3, 21–24]. The difference between our findings and those of published reports might be explained by distinctive study design, as ours assessed cytokine response to TLR ligands under different vitamin D concentrations, and not cytokine response to direct vitamin D stimulation. Because direct vitamin D supplementation deemed to increase IL-10 production, our result suggested a reduced IL-10 response to TLR stimulation with higher vitamin D concentration. Similar result was observed in the study of Belderbos, in which they found high concentration of 1,25-OHD to suppress IL-10 response to LPS stimulation in adult PBMC [25]. As a potent anti-inflammatory immune modulator, studies have shown vitamin D to downregulate TLR expression in monocytes resulting in reduced downstream cytokine production [11, 13]. Thus, we speculated that, with increasing vitamin D level, the ability of TLR to trigger IL-10 production is diminished when compared to lower concentration, thus displaying a negative correlation between vitamin D level and cytokine response to TLR stimulation. In addition, studies have demonstrated higher vitamin D levels at birth to be associated with lower number of T regulatory (Treg) cells in the cord blood [2, 16]. Since one potential source of IL-10 is Treg, it was speculated that higher vitamin D level would be associated with less Treg cells to respond to TLR stimulation, thus resulting in lower IL-10 response as seen in our study. Further research is warranted to investigate the mechanism in which maternal vitamin D status affects neonatal TLR-related IL-10 response.

Evidences have shown low production of IL-10 at birth to be strongly associated with susceptibility to acute respiratory tract infections in children aged 5 years [26]. However, in present analyses, albeit an association between maternal vitamin D level and viral TLR-triggered IL-10 response, there was no effect on the prevalence of infectious diseases during the first 12 months of life. Our observations are at odds with several studies that showed lower vitamin D status to be associated with increased incidence or severity of infection during early childhood. However, most studies that showed protective effect of vitamin D against respiratory diseases were of interventional studies that used supplementary vitamin D and did not measure serum vitamin D level. Although the study of Belderbos et al. demonstrated an association between low cord blood vitamin D level and increased respiratory syncytial virus infections in infancy, however, unlike our participants, around 50% of their neonates were vitamin D sufficient [27–29]. Since the majority of our participants were vitamin D deficient, it is possible that higher serum levels might be required to reach optimal protective effect to result in significant clinical differences. Nonetheless, in support of our results, several studies also failed to show a difference in serum vitamin D level between children with and without respiratory infections [30–32]. These inconsistent observations point to the complicated role of vitamin D in the immune modulation and disease process. The null results from our observation suggested that since the immune system is composed of multiple cells and variable pathways, having effect on only certain cytokines, such as IL-10, might not have an overall effect on disease outcome.

Our study had several limitations. First, the predominantly low serum level of 25(OH)D in this study has limited our ability to determine whether there is an association between higher concentration of vitamin D and infection. It has also limited the applicability of our result in representing the general population, although inadequate vitamin D concentration seems particularly common among pregnant woman [3, 19]. In addition, having a majority of population with suboptimal vitamin D level, we were unable to perform analysis with commonly used clinical cut-offs of vitamin D (deficient < 20 ng/mL, insufficient 20–29.9 ng/mL, sufficient ≥ 30 ng/mL). Thus, future studies related to the effect of prenatal vitamin D level on neonatal innate immunity and subsequent health outcomes will need to focus on populations with more vitamin D sufficient pregnant mothers. In addition, because only cultured supernatants were harvested in this study, we did not perform tests on T regulatory cells or maturation of the monocytes. Thus, our data could not provide detailed mechanism on how maternal vitamin D level affected neonatal TLR-triggered IL-10 response. Finally, although present study had observed an association between prenatal vitamin D status and viral TLRs-triggered response (TLR3 and TLR7/8, both of which recognize viral RNA), the effect of vitamin D on CpG double stranded DNA motif (TLR9) of the viral genome also demands further exploration. However, due to technical issues, analysis was only completed in very few participants. The result showed no correlation between maternal vitamin D status and neonatal TLR9 triggered cytokine response (Supplement 2), though such conclusion needed to be interpreted with caution for the null correlation might be due to lack of power owing to small number of test samples.

In conclusion, our study had shown that maternal vitamin D level, but not cord vitamin D, was associated with TLR3 and TLR7-8 triggered IL-10 response. This study emphasized that even though cord vitamin D level was strongly correlated with maternal vitamin D status, the extent of impact on fetal immune cytokine response might be distinctive. Although our study did not show maternal vitamin D concentration to have a significant impact on the magnitude or functional capacity of the young infant to defend against infection, we believe our study may contribute to a better understanding of the effect of prenatal vitamin D status on neonatal innate immunity and infectious disease during early life.

## Supplementary Material

Supplement 1. Maternal peripheral blood monocytes were collected and stimulated with TLR ligands to test for the expression of TNF-α, IL-6, and IL-10. Regression analysis was used to determine the relation between maternal blood 25(OH)D concentration and TLR-induced cytokine response as continuous variables. Preliminary result showed no correlation between maternal vitamin D status and cytokine response to TLR ligands in maternal mononuclear cells.
Supplement 2. Cord blood monocytes were collected and stimulated with TLR9 ligand to test for the expression of TNF-α, IL-6, and IL-10. Regression analysis was used to determine the relation between cord 25(OH)D concentration and TLR9 -induced cytokine response. Preliminary result showed no correlation between cord vitamin D status and neonatal TLR9-triggered cytokine response.

## Figures and Tables

**Table 1:** Characteristics of mother-neonatal pairs at delivery.

Characteristics	Data
*Mothers* (*n*)	372
Age at enrollment (y)	29.4 (28.6–30.1)
History of allergy	129 (34.8)
Smoking during pregnancy	35 (9.5)
Education	
Primary or secondary	11 (3.0)
High school	99 (26.6)
College or above	262 (70.4)
Mode of delivery (NSD)	234 (62.9)
25(OH)D (ng/mL); median (IQR)	15.18 (10.85–19.10)
*Neonates* (*n*)	372
Sex (male)	179 (48.6)
BBW (g)	3087 ± 481
Gestational age (weeks)	38.3 ± 1
Season of birth	
Spring	107 (28.8)
Summer	105 (28.2)
Autumn	88 (23.7)
Winter	68 (18.3)
25(OH)D (ng/mL); median (IQR)	14.80 (10.02–18.86)

Values are listed as *n* (%) or mean ± SD, as appropriate.

NSD: natural spontaneous delivery.

IQR: interquartile.

**(a):** 

	Univariate analysis β (95% CI)	*p*	Multivariate analysis *β* (95% CI)	*p*
*TLR1-2*				
TNF-*α*	0.42 (0.44, 1.47)	0.36	0.55 (−0.53, 1.59)	0.30
IL-6	0.35 (−0.85, 0.79)	0.92	0.13 (−0.79, 1.05)	0.79
IL-10	0.04 (−0.79, 0.86)	0.93	0.12 (−0.82, 1.06)	0.76
*TLR3*				
TNF-*α*	0.15 (−0.64, 0.96)	0.73	0.15 (−0.68, 0.94)	0.93
IL-6	−0.45 (−1.15, 0.33)	0.26	−0.42 (−1.48, 0.54)	0.40
IL-10	−0.85 (−1.45, −0.24)	0.007	−1.05 (−1.70, −0.34)	0.004
*TLR4*				
TNF-*α*	0.12 (−0.19, 0.50)	0.43	0.08 (−0.28, 0.44)	0.44
IL-6	−0.17 (−0.46, 0.12)	0.27	−0.24 (−0.54, 0.08)	0.14
IL-10	−0.03 (−0.16, 0.11)	0.69	0.19 (−0.55, 0.15)	0.28
*TLR7-8*				
TNF-*α*	−0.35 (−0.84, 0.12)	0.64	−0.31 (−0.83, 0.20)	0.19
IL-6	−0.05 (−0.43, 0.28)	0.79	−0.18 (−0.59, 0.21)	0.89
IL-10	−0.85 (−1.47, −0.22)	0.008	−0.99 (−1.70, −0.30)	0.006
*PHA*				
TNF-*α*	0.52 (−0.59, 1.73)	0.39	0.30 (−1.07, 1.55)	0.39
IL-6	0.52 (−0.55, 1.62)	0.33	0.30 (−1.02, 1.59)	0.67
IL-10	−0.36 (−0.34, 1.14)	0.35	0.08 (−0.80, 0.96)	0.87

Adjusted for gestational age, gender, birth body weight, mode of delivery, maternal allergy, and season of birth.

**(b):** 

	Univariate analysis β (95% CI)	*p*	Multivariate analysis β (95% CI)	*p*
*TLR1-2*				
TNF-*α*	0.63 (−0.06, 1.50)	0.09	0.73 (−0.17, 1.64)	0.12
IL-6	0.21 (−0.63, 1.20)	0.67	0.51 (−0.77, 1.51)	0.51
IL-10	0.21 (−0.74, 1.11)	0.62	0.51 (−0.68, 1.50)	0.41
*TLR3*				
TNF-*α*	0.77 (−0.01, 1.55)	0.06	0.63 (−0.27, 1.50)	0.16
IL-6	−0.13 (−0.83, 0.63)	0.73	0.18 (−0.86, 1.18)	0.71
IL-10	−0.17 (−0.67, 0.41)	0.54	−0.18 (−0.83, 0.50)	0.62
*TLR4*				
TNF-*α*	0.20 (−0.22, 0.72)	0.41	0.21 (−0.41, 0.69)	0.70
IL-6	−0.05 (−0.38, 0.26)	0.77	0.01 (−0.39, 0.39)	0.96
IL-10	−0.03 (−0.16, 0.11)	0.69	0.19 (−0.55, 0.15)	0.28
*TLR7-8*				
TNF-*α*	0.14 (−0.29, 0.58)	0.55	0.39 (−0.05, 0.85)	0.95
IL-6	0.14 (−0.36, 0.60)	0.60	−0.03 (−0.48, 0.47)	0.89
IL-10	−0.51 (−1.33, 0.23)	0.16	−0.49 (−1.64, 0.52)	0.40
*PHA*				
TNF-*α*	0.94 (−0.06, 2.02)	0.78	0.61 (−0.45, 1.79)	0.29
IL-6	0.94 (−0.10, 2.00)	0.08	0.61 (−0.47, 1.74)	0.30
IL-10	0.56 (−0.27, 1.34)	14	0.47 (−0.56, 1.34)	0.30

Adjusted for gestational age, gender, birth body weight, mode of delivery, maternal allergy, and season of birth.

**Table 3:** Association of maternal and cord serum 25(OH)D levels with clinical outcome at 1 year of age.

	Univariate	*p*	Multivariate	*p*
	OR (95% CI)		OR (95% CI)	
*Maternal serum 25(OH)D*				
Bronchiolitis	1.26 (0.47, 3.37)	0.65	1.25 (0.42, 3.71)	0.69
Pneumonia	2.57 (0.41, 16.07)	0.31	4.58 (0.52, 40.51)	0.17
Croup	7.10 (0.85, 60.81)	0.07	13.29 (0.88, 199.8)	0.06
Enteritis	1.20 (0.36, 4.02)	0.77	1.47 (0.38, 5.62)	0.58
UTI	0.85 (0.25, 2.94)	0.80	0.92 (0.26, 3.24)	0.92
*Cord serum 25(OH)D*				
Bronchiolitis	2.22 (0.90, 5.47)	0.08	1.13 (0.42, 3.04)	0.81
Pneumonia	1.70 (0.36, 7.94)	0.51	2.38 (0.22, 25.46)	0.47
Croup	3.82 (0.67, 21.81)	0.13	4.64 (0.75, 28.80)	0.10
Enteritis	0.98 (0.36, 2.69)	0.97	1.42 (0.43, 4.69)	0.56
UTI	0.75 (0.25, 2.29)	0.62	0.78 (0.25, 2.45)	0.67

Total number of children aged 1 year: 321.

Number of bronchiolitis: 45 (14%), pneumonia: 11 (3.4%), croup: 8 (2.5%), infectious enteritis: 22 (6.9%), and UTI (urinary tract infection): 27 (8.4%).

Adjusted for gestational age, sex, birth body weight, mode of delivery, season of birth, and maternal allergy.
